# Chromosome aneuploidy analysis in embryos derived from in vivo and in vitro matured human oocytes

**DOI:** 10.1186/s12967-021-03080-1

**Published:** 2021-10-09

**Authors:** Jianhua Li, Jing Chen, Tiecheng Sun, Shuiwen Zhang, Tingting Jiao, Ri-Cheng Chian, Youzhu Li, Ye Xu

**Affiliations:** 1 Reproductive Medical Center, Senior Department of Obstetrics & Gynecology, The Seventh Medical Center of PLA General Hospital, Beijing, 100700 China; 2 Reproductive Medicine Center, The First Affiliated Hospital of Xiamen University, No. 6 Guchengxi Road, Si Ming, Xiamen, 361003 China; 3 Research Group for Reproductive Medicine and IVF Laboratory, Department of Obstetrics and Gynecology, Cologne University, Kerpener Str. 7, 50931 Cologne, Germany; 4 Reproductive Medical Center, Department of Obstetrics and Gynecology, Peking University International Hospital, Beijing, 102206 China; 5 Center for Reproductive Medicine, Shanghai Tenth People’s Hospital of Tongji University, Shanghai, 200072 China

**Keywords:** In vitro oocyte maturation (IVM), In vitro fertilization (IVF), Embryo, Aneuploidy, Preimplantation genetic screening (PGS)

## Abstract

**Background:**

In vitro oocyte maturation (IVM) is being increasingly approached in assisted reproductive technology (ART). This study aimed to evaluate the quality of embryos generated by in-vitro matured immature follicles, as a guideline for further clinical decision-making.

**Methods:**

A total of 52 couples with normal karyotypes underwent in vitro fertilization, and 162 embryos were donated for genetic screening. Embryos in IVF group were generated by mature follicles retrieved during gonadotrophin-stimulated in vitro fertilization (IVF) cycles. And embryos in IVM group were fertilized from IVM immature oocytes.

**Results:**

The average age of the women was 30.50 ± 4.55 years (range 21–42 years) with 87 embryos from IVF group and 75 embryos from IVM group. The rate of aneuploid with 28 of the 87 (32.2%) embryos from IVF group and 21 of the 75 (28%) embryos from IVM group, with no significant difference. The frequency of aneuploid embryos was lowest in the youngest age and increased gradually with women’s age, whether in IVF group or IVM group and risen significantly over 35 years old. The embryos with morphological grade 1 have the lowest aneuploidy frequency (16.6%), and increase by the grade, especially in IVF group. In grade 3, embryos in IVM group were more likely to be euploid than IVF group (60% vs 40%, respectively).

**Conclusions:**

IVM does not affect the quality of embryos and does not increase the aneuploidy rate of embryos. It is clinically recommended that women more than 35 years have a high aneuploidy rate and recommended to test by PGS (strongly recommended to screened by PGS for women more than 40 years). Women aged less than 35 years old for PGS according to their physical and economic conditions. Embryo with poor quality is also recommended to test by PGS, especially for grade III embryos.

## Background

A growing number of couples adopt assisted reproductive technologies (ART) in infertility clinics to conceive a child. Due to the dominant follicles in follicular development may suppress the small follicles’ growth, nearly 15% of oocytes retrieved by gonadotrophin-stimulated in vitro fertilization (IVF) cycles are immature [1], and these immature oocytes are usually discarded. However, in controlled ovarian hyperstimulation (COH) cycle high yield of follicle may increase the risk of complications, such as: ovarian hyperstimulation syndrome (OHSS), torsion of ovarian pedicle, and intra-abdominal bleeding [2]. Furthermore, Baart et al. suggested that high-dose of gonadotrophin-stimulated in COH cycle may lead to high frequency of aneuploidy [3]. In vitro maturation (IVM) of immature follicles as an alternative method which has become a promising strategy to maximize the utilization of follicles and maximum cost savings, it could be beneficial for women with polycystic ovaries, poor ovarian response to gonadotropin treatment, premature ovarian failure, and cryopreserve their oocytes before monotherapy to preserve their fertility [4].

The procedure and the techniques used for IVM is no generally accepted and substantially different across infertility clinics. The quality of mature oocyte is the key to embryo quality and developmental potential leading to chromosomal anomalies or embryo loss. Therefore, ensuring couples conceive in decrease time with healthy embryo is important through genetic analysis of embryo for reproductive medicine. For human embryos, aneuploidy is of great relevance to embryo selection. The most common type of genetic abnormality and the leading cause of miscarriage, implantation failure is aneuploidy [5]. More than half of embryos produced by IVF cycles are aneuploid [6].

IVM of immature oocytes, recovered from natural cycle, is a useful cost-effective treatment for infertility in women. Whereas, there was no detectable comprehensive evidence on the frequency of aneuploidy on these embryos based on IVM of immature oocytes retrieval. As well, the relationship between genetic status and embryo morphology remains unclear. Preimplantation genetic screening (PGS) for embryos has gradually become a gold criterion in infertility treatment, offer a more accurate assessment of chromosome status, not only to enhance the implantation and pregnancy rates, but also to improve the healthy offspring, such as: reduce miscarriages, risk of aneuploid offspring, and time to conceive. We aimed to compare the quality of embryos between IVM and IVF groups through genetic screening, for further clinical decision-making.

## Materials and methods

### Study design and patients

This study was reviewed and approved by the Ethics Committee at The Seventh Medical Center of PLA General Hospital (Research License 2021-31). All embryos were obtained from donors at the Center of Reproductive Medicine in The Seventh Medical Center of PLA General Hospital. Recruitment took place from April 2005 to February 2015.

Written informed consent was provided before donating their surplus frozen embryos for research. The donors were financially compensated for the effort, time and inconvenience related to the donation process. A total of 162 embryos donated from 52 couples with normal karyotypes were used in this study. All of the women with normal ovaries, uterus, and regular menstrual cycles had normal basal FSH levels (< 10 mIU/mL on day 3 of the menstrual cycle). We performed preimplantation genetic screening (PGS) for chromosome aneuploidy analysis in embryos derived from in vivo (IVF group) and in vitro matured human (IVM group) oocytes.

### Controlled ovarian hyperstimulation (COH) in IVF group

For patients with COH cycle, the treatment was initiated from a baseline transvaginal ultrasound scan on day 3 of the menstrual cycle to ensure that there were more than seven small antral follicles in both ovaries. Ovary stimulation was carried out with exogenous gonadotropins after a desensitization protocol with GnRH analogues according to a long or a short protocol, depending on the patient’s previous history of gonadotropin response and other factors. An ultrasound scan was performed on day 7–9 and subsequently until one follicle reached 18 mm and two reached 16 mm in diameter, and then 10,000 IU human chorionic gonadotropin (hCG) was administered. Oocyte retrieval was performed approximately 36 h after hCG administration.

### Follicle retrieval and in-vitro maturation (IVM)

IVM is routinely performed in our center for patients who had normal ovaries with > 7 antral follicles in both ovaries. Briefly, the treatment was initiated from a baseline transvaginal ultrasound scan on day 3 of the menstrual cycle to ensure that there were more than seven small antral follicles in both ovaries. On day 7–9 ultrasound scans were repeated. When the dominant follicle reached 12–14 mm in diameter and/or the endometrial thickness was ≥ 6 mm, hCG (10,000 IU) was administered and oocyte retrieval was performed 36 h later.

The oocyte retrieval was performed using transvaginal ultrasound-guided aspiration was performed with a 17-gauge double-lumen needle (Cook, Eight Mile Plains, Queensland, Australia) for aspiration of the leading follicles. A 19-gauge single-lumen needle (Cook) for the small follicles. For aspiration, a portable pump was connected to the needle with a pressure of < 100 mm Hg for leading follicles and < 40 mm Hg for small follicles. After assessing the nuclear maturity of the retrieved oocytes under the dissecting microscope as the commonly used method [7]. The collected matured oocytes were inseminated 2 or 3 h later by intracytoplasmic sperm injection (ICSI), while the collected immature oocytes were cultured in IVM medium.

The immature oocytes at Metaphase-I (MI) and GV stage were cultured in a 1 mL maturation medium containing 30% serum of the patient’s own (inactivated at 56° for 30 min) with 75 mIU/mL human FSH ((Gonal-F; Merck Serono, Switzerland), 75 mIU/ml human menopausal gonadotrophin (hMG, Lizhu Pharmacy) and 10 ng/ml recombinant human epidermal growth factor (Invitrogen, Carlsbad, CA, USA) to induce final oocyte maturation at 37 °C in 5% CO_2_, 5% O_2_ and 90% N_2_ with high humidity for 24 or 48 h. Then the oocytes matured in vitro were also inseminated by ICSI [8].

### Embryo culture

All embryos were fertilized using ICSI. The zygotes were cultured in individual 20-µL droplets of G1-PLUS medium (Vitrolife, Gothenburg, Sweden) overlaid with 2.5-mL mineral oil (Vitrolife, Gothenburg, Sweden) in a 30-mm Falcon culture dish and incubated at 37 °C in an atmosphere containing 5% O_2_ and 6.0% CO_2_. On day 3 or day 4 oocyte retrieval, the embryos were vitrification for the further genetic screen.

### Whole-genome amplification and DNA sequencing

After thawing, whole embryo (WE) samples were transferred into RNase- and DNase-free PCR tubes containing 5 μL cell lysis buffer (Yikon Genomics, China), and frozen immediately in liquid nitrogen stored at − 80 °C until further processing.

DNA for whole-genome amplification was amplified with the multiple annealing and looping based amplification cycles (MALBAC) technique and library generation (Cat No. YK001B, Yikon Genomics) as previously described [9, 10]. Amplification products were sequenced on an Illumina HiSeq 2500 platform (Illumina, San Diego, CA, USA) with approximately two million sequencing reads per sample. The read numbers were counted along the whole genome with a bin size of 1 Mb and normalized based on GC content and a reference dataset. The number of reading counts served as the index of ploidy: a 50% increase indicates an increase in the number of chromosomes from 2 to 3, whereas a 50% decrease indicates a reduction in the number of chromosomes from 2 to 1 [11, 12].

### Statistical analysis

Continuous data were reported based on mean ± standard deviations. Data analysis was performed using SPSS (version 20). The frequency between the groups were assessed using Fisher’s exact test. Differences were considered significant at P < 0.05.

## Results

**Fig. 1:**
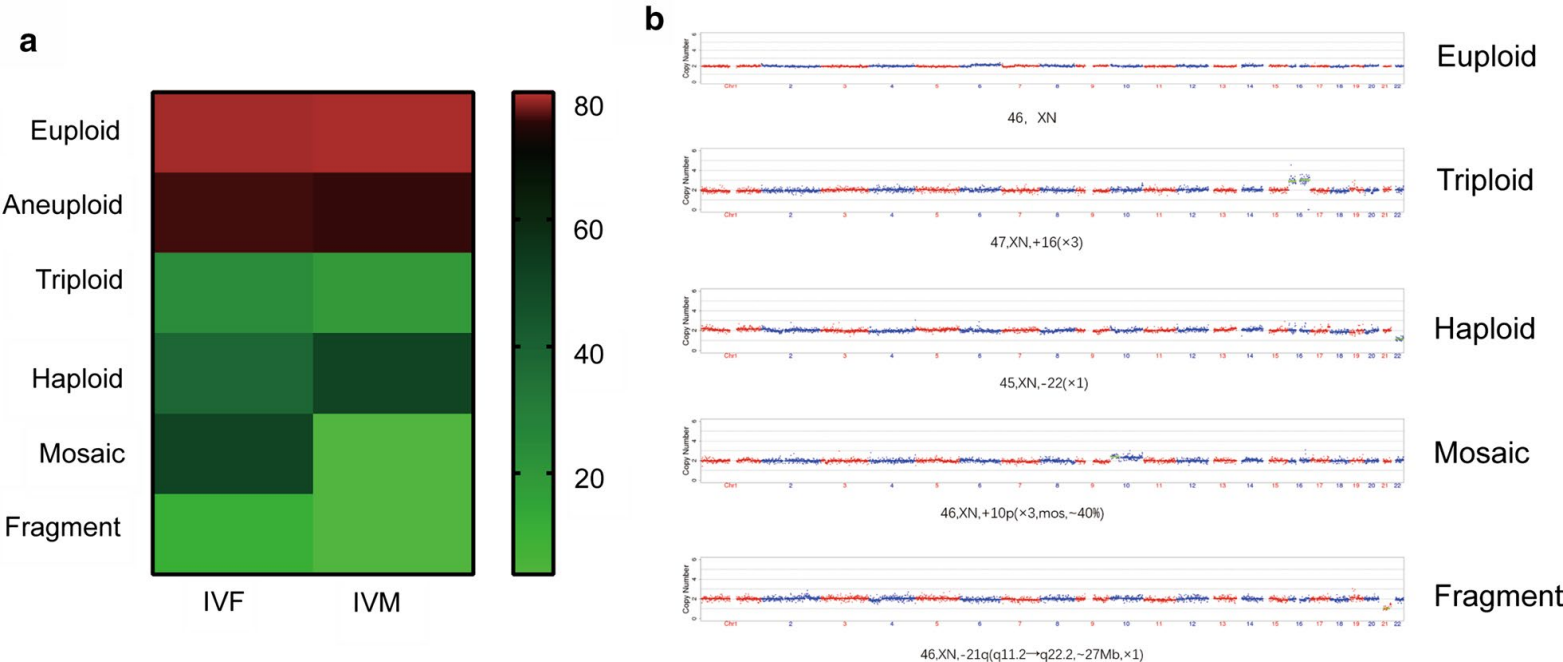
Clinical characteristics of patients for whole embryos PGS between IVF group and IVM groups. **a** Heatmap of whole embryos PGS in aneuploidy rates (%) between IVF group and IVM groups. **b** The demonstration of whole embryos PGS profiles for embryos between IVF group and IVM groups.

A total of 162 embryos were recovered and analyzed for PGS from 53 couples. The average age of the women was 30.50 ± 4.55 years (range 21–42 years) with 87 embryos from IVF group and 75 embryos from IVM group. There was no significant difference (P > 0.05) between the two groups in euploid or aneuploid. The rate of aneuploid with 28 of the 87 (32.2%) embryos from IVF group and 21 of the 75 (28%) embryos from IVM group (Table 1; Fig. 1). Only 5.7% triploid, 6.9% haploid, 8.0% mosaic, 4.6% fragment abnormality, and 10.3% chaotic in 87 embryos samples of IVF group vs. only 5.5% triploid, 8.0% haploid, 4.0% mosaic, 4.0% fragment abnormality, and 13.3% chaotic were also detected in 75 embryos samples of IVM group (Table 1; Fig. 1).

**Table 1:** Clinical characteristics of patients for whole embryos PGS between IVF group and IVM groups

	IVF	IVM	Total
Number of patients	26	26	52
Age (Year)	30.96 ± 5.74	30.07 ± 3.13	30.50 ± 4.553
Number of embryo sample	87	75	162
Euploid (%)	59 (67.8)	53 (70.7)	112 (69.1)
Aneuploid (%)	28 (32.2)	21 (28.0)	59 (36.4)
Triploid (%)	5 (5.7)	4 (5.3)	9 (5.6)
Haploid (%)	6 (6.9)	6 (8.0)	12 (7.4)
Mosaic (%)	7 (8.0)	3 (4.0)	10(6.2)
Fragment (%) Abnormality (%)	4 (4.6)	3 (4.0)	7 (4.3)
Chaotic (%)	9 (10.3)	10 (13.3)	19 (11.7)

Values shown are n, n (%) or mean ± SD, unless otherwise noted

### Correlation of age with euploidy

**Fig. 2:**
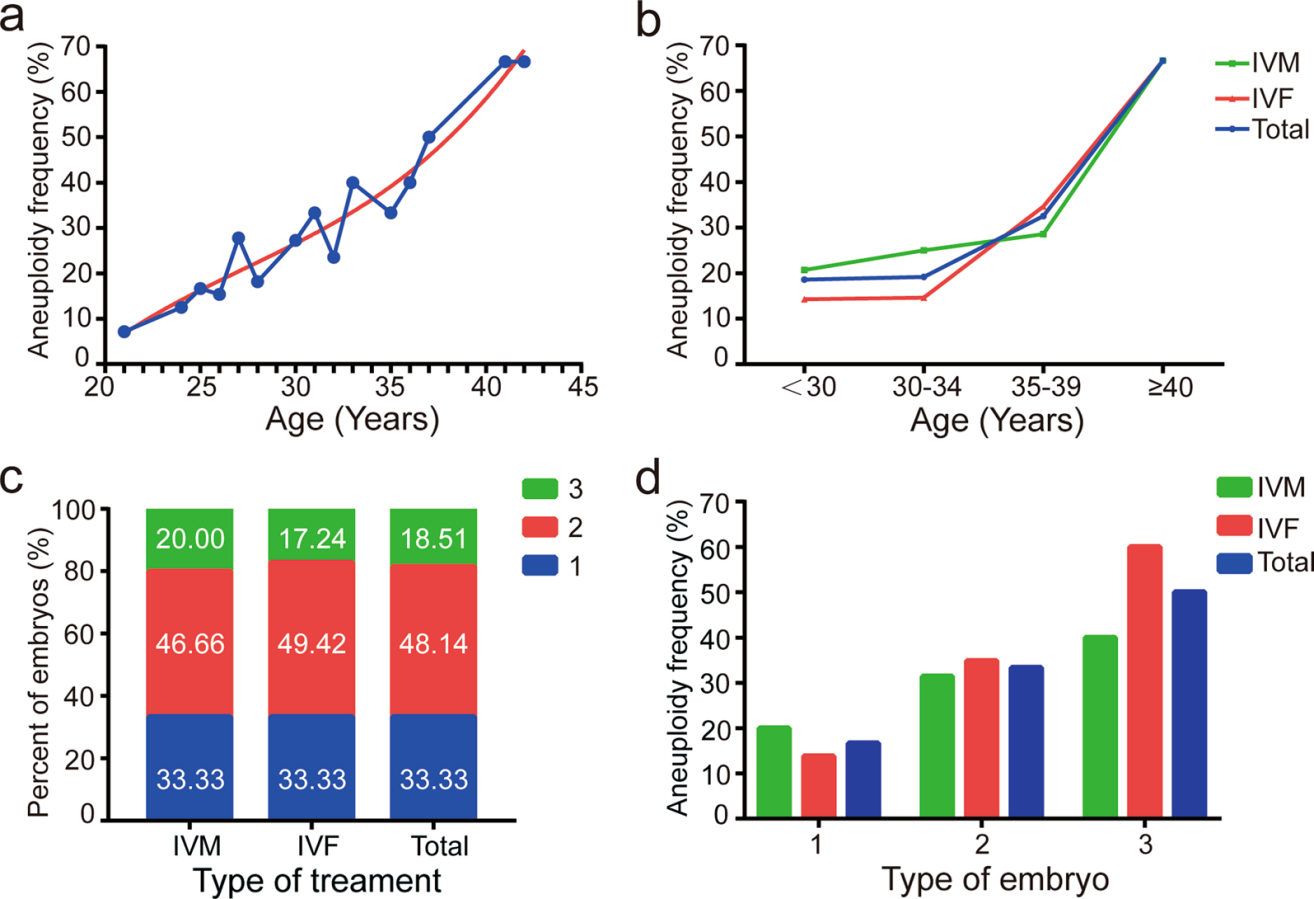
Embryo aneuploidy rate according to the woman’s age and morphology grade of the embryo. **a** The association between women’s age and aneuploidy rates. Aneuploidy rates are lowest in young women and start to gradually increase with age. **b** Embryo aneuploidy rates in different age stages of women between IVF group and IVM group. No significant differences in the aneuploid rate detected between IVF group or IVM group. **c** The percentage in each grade of the embryo on morphology between the two groups was no significant difference. **d** The worse the embryo grade, the higher the aneuploidy rate, especially in IVF group

The rate of aneuploid embryos was lowest in the youngest age and increased gradually with women’s age (Fig. 2a) Whether in IVF group or IVM group and risen significantly over 35 years old (Fig. 2b). Women below the age of 35 years are nearly 20%, increasing to 32.5% in 39 years old, and 66.66% for women more than 40 years. Notably, no significant differences in the aneuploid rate detected between IVF group or IVM group (P > 0.05).

### Correlation of embryo morphology with euploidy

The percentage in each grade of the embryo between the two groups was no significant difference (P > 0.05) (Fig. 2c). But the worse the embryo grade, the higher the aneuploidy rate, especially in IVF group (Fig. 2d). Embryos with grade 1 and 2 have a similar rate of aneuploidy both in IVM group and IVF group, but in grade III, embryos in IVM group were more likely to be euploid than IVF group (60% vs 40%, respectively). Of the 29 embryos in IVF group assigned morphology grade 1, 13.79% were aneuploid while in IVM group (25 embryos) 20% were aneuploid; of the 43 embryos with morphology grade 2, 34.88% were aneuploid in IVF group while 31.42% aneuploid of the 35 embryos in IVM group; and 60% aneuploid of the 15 embryos with grade 3 in IVF group while 40% aneuploid of the 15 embryos in IVM group (Table 2).

**Table 2:** Correlation of embryo morphology grade with chromosome aneuploidy

	IVF	IVM
A		
No. of embryos	29	25
Euploid (%)	25 (86.2)	20 (80.0)
Aneuploid (%)	4(13.79)	5 (20)
Triploid (%)	0	0
Haploid (%)	3 (10.3)	0
Mosaic (%)	1 (3.4)	1 (4.0)
Fragment abnormality (%)	1 (3.4)	1 (4.0)
Chaotic (%)	0	3 (12.0)
B		
No. of embryos	43	35
Euploid (%)	28 (65.1)	24 (68.6)
Aneuploid (%)	15 (34.88)	11 (31.42)
Triploid (%)	5 (11.6)	4 (11.4)
Haploid (%)	1 (2.3)	5 (14.3)
Mosaic (%)	1 (2.3)	2 (5.7)
Fragment abnormality (%)	3 (7.0)	2 (5.7)
Chaotic (%)	6 (14.0)	3 (8.6)
C		
No. of embryos	15	15
Euploid (%)	6 (40.0)	9 (60.0)
Aneuploid (%)	9 (60)	6 (40)
Triploid (%)	0	0
Haploid (%)	2 (13.3)	1 (6.7)
Mosaic (%)	2 (13.3)	0
Fragment abnormality (%)	3 (20.0)	0
Chaotic (%)	3 (20.0)	4 (26.7)

A, embryos of Grade 1 evaluated by morphology; B, embryos of Grade 2 evaluated by morphology; C, embryos of Grade 3 evaluated by morphology

## Discussion

Recently considerable changes are afoot regarding ‘‘routine’’ IVF. As an increasingly interesting treatment, IVM becomes attracting attention. Primarily because the mature follicles can be directly ICSI for fresh embryo transfer; particularly the immature follicles can be cultured in vitro to increase the utilization rate of follicles; finally, the occurrence of ovarian hyperstimulation syndrome can be reduced, so that patients can improve the yield rate of follicles and also reduces the side effect of repeated ovarian stimulation with GnRH and gonadotropin treatment.

In the natural ovulatory cycle, small follicles are destined to be atretic with only a dominant follicle achieve maturity and ovulation. In the conventional IVF cycle, the daily use of gonadotrophins can induce many follicular developments, but dominant follicles in ovarian stimulation would also suppress other subordinate follicles’ growth and induce atresia. Furthermore, studies have also reported that high high-dose exogenous gonadotropins cause chromosomal abnormalities and high aneuploidy rates while stimulating nuclear maturation [3, 13].

Although, IVM of immature follicles using less drug stimulation, less cost and more patient-friendly approach compared with conventional COH in IVF cycle. Recovery of these immature oocytes followed by IVM is still not clear, whether these atresia follicles can be recovered by culturing in vitro or have the ability of folliculogenesis again? Whether the embryos formed by these in vitro mature follicles are healthy or not?

Latest studies in animal models show that the dominant follicle does not adversely affect the developmental and maturational competence of immature bovine oocytes [14], this conclusion has also been verified in human that the development of immature oocytes is not affected either by the presence of a dominant follicle or by the phase of folliculogenesis [15], the results were similar with ours. We performed with 87 embryos from 26 IVF women and 75 embryos from 26 IVM women, the aneuploid rate is higher in IVF embryos, (32.2% in IVF vs 28.0% in IVM, respectively) with no significant difference (Table 1). This result was similar to a previous retrospective study in that the frequency of chromosomal abnormality in cleavage stage embryos from IVM and IVF cycles were no significant different [16]. This may be due to the supraphysiological levels of some growth factors and hormones in IVM media, and the less competition for follicles to get these factors in vitro to compare to culture in vivo [17]. A similar result in another study suggested that women with premature ovarian insufficiency using IVM of immature follicles can get higher pregnancy and delivery rate and yield better clinical outcomes [18].

We survey the analysis of the aneuploidy frequency in combination with female age showed that aneuploidy rate was increased gradually with women’s age. Women below the age of 35 years are nearly 20%, increasing to 32.5% in 39 years old, and 66.66% for women more than 40 years. This is similar to the study from Fragouli et al. [19]. Women reaching age 35 years face a decline in fecundability attributed to the steep increase in aneuploidy rates which is reflected by the higher miscarriage rates [6, 20].

In this study, the embryos with morphological grade 1 have the lowest aneuploidy frequency (16.6%) and increase by the grade (Fig. 2d). This was similar to previous studies that observed that the embryos with good quality were more likely to be euploid [21]. Embryos with grade 1 and 2 have a similar rate of aneuploidy both in IVM group and IVF group, but in grade III, embryos in IVM group were more likely to be euploid than IVF group (60% vs 40%, respectively). Due to the pathway in immature follicles were continue to express as well as the expression of genes related to cellular and homeostasis storage, IVM medium has supplementation of the factor for oocyte maturation than in vivo [22].

In conclusion, the results from this study demonstrate that IVM does not affect the quality of embryos, also does not increase the aneuploidy rate of embryos. For women, more than 40 years have a high aneuploidy rate and need to be screened by PGS. An embryo with poor quality also needs to be tested by PGS.

## Data Availability

The datasets used and analyzed during the current study are available from the corresponding author on reasonable request.

## Abbreviations

ART: Assisted reproductive technology
IVF: In vitro fertilization
IVM: In vitro oocyte maturation
PGS: Preimplantation genetic screening
ICSI: Intracytoplasmic sperm injection
COH: Controlled ovarian hyperstimulation
OHSS: Ovarian hyperstimulation syndrome
hMG: Human menopausal gonadotrophin
